# GPU Ray Tracing for the Analysis of Light Deflection in Inhomogeneous Refractive Index Fields of Hot Tailored Forming Components

**DOI:** 10.3390/s25061663

**Published:** 2025-03-07

**Authors:** Pascal Kern, Max Brower-Rabinowitsch, Lennart Hinz, Markus Kästner, Eduard Reithmeier

**Affiliations:** Institute of Measurement and Automatic Control, Stiftung Gottfried Wilhelm Leibniz Universität Hannover, An der Universität 1, D-30823 Garbsen, Germany; max.brower@imr.uni-hannover.de (M.B.-R.); lennart.hinz@imr.uni-hannover.de (L.H.); markus.kaestner@imr.uni-hannover.de (M.K.); eduard.reithmeier@imr.uni-hannover.de (E.R.)

**Keywords:** optimal simulation, light deflection, ray tracing, uncertainty simulation, inhomogeneous refractive index fields, tailored forming

## Abstract

In hot-forming, integrating in situ quality monitoring is essential for the early detection of thermally induced geometric deviations, especially in the production of hybrid bulk metal parts. Although hybrid components are key to meeting modern technical requirements and saving resources, they exhibit complex shrinkage behavior due to differing thermal expansion coefficients. During forming, these components are exposed to considerable temperature gradients, which result in density fluctuations in the ambient air. These fluctuations create an inhomogeneous refractive index field (IRIF), which significantly affects the accuracy of optical geometry reconstruction systems due to light deflection. This study utilizes existing simulation IRIF data to predict the magnitude and orientation of refractive index fluctuations. A light deflection simulation run on a GPU-accelerated ray tracing framework is used to assess the impact of IRIFs on optical measurements. The results of this simulation are used as a basis for selecting optimized measurement positions, reducing and quantifying uncertainties in surface reconstruction, and, therefore, improving the reliability of quality control in hot-forming applications.

## 1. Introduction

Hot-forming processes are key in producing high-performance components exposed to high mechanical and thermal loads [1,2]. In this context, hybrid components combine materials with different properties and are also increasingly used. This realizes modern technical applications while saving resources [3]. Due to the short cycle times of hot-forging processes, rejects can only be detected relatively late and eliminated by making adjustments to the process, which means that many faulty components are already being produced. A high reject rate significantly impacts the economic efficiency and sustainability of the methods as both material and energy are used to heat the semi-finished products and produce the rejected parts. Consequently, integrating metrological processes for in situ quality control is particularly important [4].

**Figure 1:**
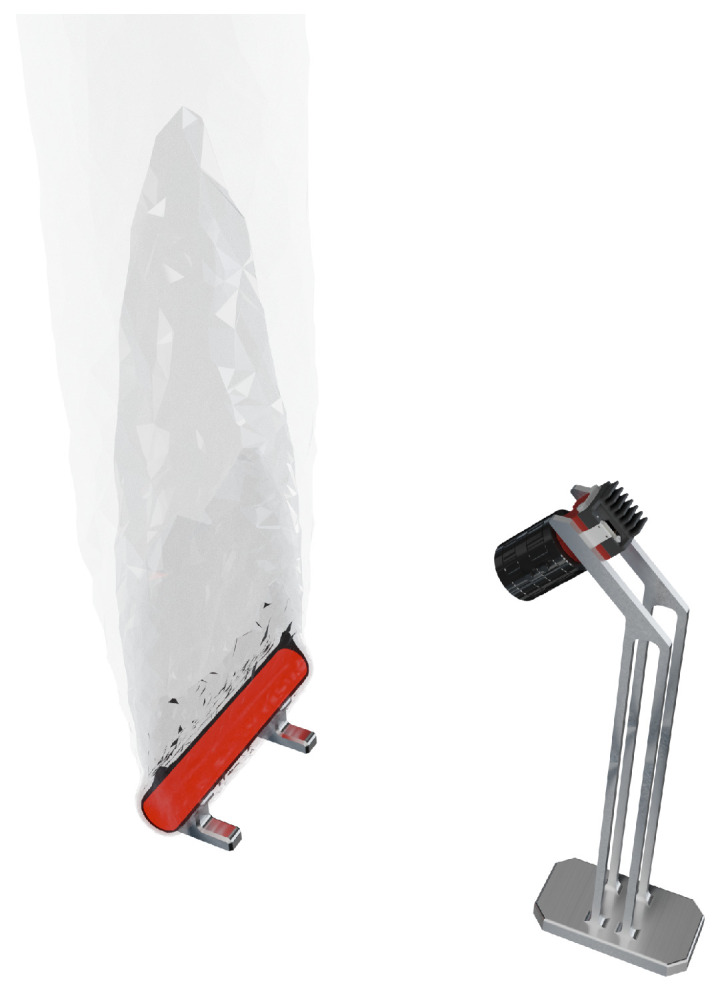
A camera records a hot measurement object and is influenced by an inhomogeneous refractive index field.

In recent years, various commercial systems for optical geometry reconstruction have been established in industrial applications, such as those based on fringe projection profilometry. Due to self-emission and the heat input into the ambient air, optical measurement of the geometry of hot-forged components, often exceeding 1100 °C, represents a current metrological limit. The heating of the surrounding air induces density fluctuations, culminating in an IRIF [5]. This is visualized in Figure 1, where an inhomogeneous refractive index field is created around the hot-glowing component, which rises upwards with the hot air. The camera image will, therefore, be distorted.

The presence of an IRIF adversely affects the accuracy of optical geometry reconstruction systems that are commonly employed for quality control purposes [6]. Therefore, a comprehensive understanding of IRIFs and their impact on optical measurements is essential to mitigate measurement errors and enhance the reliability of in situ quality assessments.

Several studies have previously explored this issue. Deng et al. investigated the measurement errors in stereo camera systems caused by light refraction at high temperatures, developing a nonlinear imaging model to predict measurement inaccuracies [7]. Their approach involved simulating the air temperature distribution around high-temperature components to estimate the nonlinear refractive index distribution and deriving a model based on multiple simplifications, such as assuming planar component surfaces and symmetric viewing directions. While their findings provided valuable insights into the influence of parameters like temperature and camera baseline distance on measurement errors, their model lacked the flexibility to generalize the impact of IRIFs on complex geometries and arbitrary measurement positions, which is critical for hot-forming applications.

Beermann et al. extended this approach by presenting a comprehensive simulation model for laser triangulation in an IRIF, accounting for light deflection along the entire path from the illumination source to the object and then to the camera [5]. Although their CPU-based ray tracing approach yielded several key observations, it was constrained to a single sensor pose. The computational inefficiency inherent in CPU-based simulations impeded a global assessment of the IRIF effect across complex geometries, limiting its applicability for comprehensive analysis.

The calculation of refracted light rays is fundamental in optical systems where a known refractive index gradient modifies the direction of light rays, such as in gradient-index lenses. Montagnino was among the first to publish calculations of light paths within gradually changing refractive index fields by discretizing the refractive index distribution and numerically integrating the ray equations using Runge–Kutta methods [8]. Subsequent enhancements improved the performance, generalized the refractive index field representations, and increased the accuracy by adjusting the step sizes based on the refractive index gradients [9,10,11]. While these numerical methods offer precise calculations of light deflection effects, they are computationally intensive and, thus, less suitable for inline applications requiring rapid computations.

This study employs a GPU-accelerated ray tracing approach to address these limitations and simulate light deflection under IRIF conditions across multiple camera-efficient poses and complex geometries. Utilizing pre-established IRIF data and various component geometries from [12] simulated in COMSOL Multiphysics (Stockholm, Sweden) [13], the study aims to predict the magnitude and orientation of refractive index variations and evaluate their influence on optical measurement systems. The GPU-based simulation enables rapid computation of IRIF effects for diverse measurement scenarios, facilitating the optimization of measurement positions. Consequently, this approach significantly reduces uncertainties in surface reconstruction and enhances the reliability of in situ quality control in hot-forming processes. Our approach makes it possible to find optimal sensor poses and quantify uncertainties depending on the IRIF. The following section outlines the main theoretical framework and describes our investigation’s essential materials and methods.

## 2. Materials and Methods

**Figure 2:**
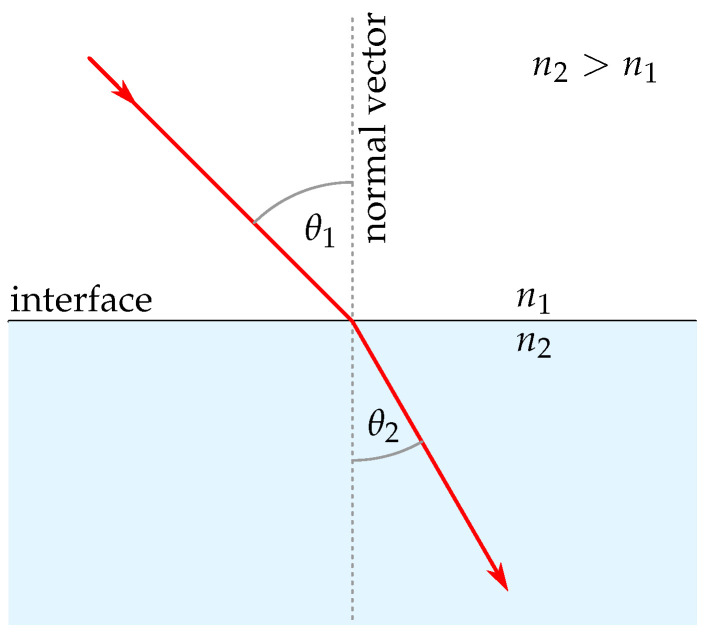
Visualization of Snell’s law of refraction.

Ray tracing in refractive media, based on geometric optics, is based primarily on Snell’s law of refraction. Independently formulated in the 17th century by Snellius, Descartes, and Harriot [14], this law describes the change in direction $\left(\theta_{1}\to \theta_{2}\right)$ of a light ray as it transitions between two homogeneous media in 3D:

(1) $$n_{1}\left(d_{i}\;\cdot \;n\right)=n_{2}\left(d_{t}\;\cdot \;n\right)$$

where $n_{1}$ and $n_{2}$ describe refractive indices of the first and second media, $d_{i}$ the incident direction vector (normalized), $d_{t}$ the transmitted (refracted) direction vector (normalized), and n the surface normal vector (normalized). Figure 2 illustrates Snell’s law of refraction.

**Figure 3:**
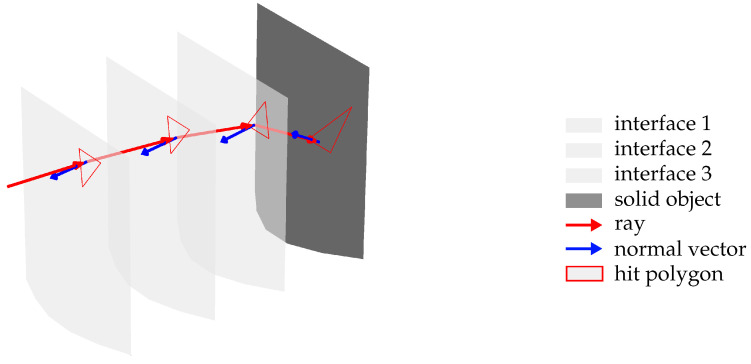
Visualization of ray tracing through an inhomogeneous refractive medium.

The main steps include determining the intersection point between the incident ray and the interface and computing the direction of the reflected or refracted ray according to the laws of reflection and refraction. Historically, ray tracing has evolved primarily to achieve realistic illumination in three-dimensional virtual environments. The physically accurate method would involve tracing all light rays emitted by light sources within the scene, considering only those rays that eventually intersect with the virtual camera to generate an image based on their intensity. However, this approach is highly inefficient and computationally demanding. To address these challenges, ray tracing systems exploit the principle of optical reciprocity of light, first described by von Helmholtz [15], which states that, in linear optical systems, the path of light is reversible; that is, the trajectory of a light ray is the same regardless of the direction in which it is traversed. As a result, calculations of reflections and refractions between two points can be performed in either direction. This principle is extensively utilized in contemporary ray tracing to produce physically accurate computer-generated renderings of three-dimensional scenes, especially in the entertainment industry [16]. By tracing rays originating from the camera rather than from the light sources, a significant reduction in computational effort is achieved [17]. Figure 3 depicts the concept of ray tracing through an inhomogeneous refractive medium.

In this methodology, the IRIF generated around the hot-forged measurement object is discretized into iso-index boundary layers based on data from the COMSOL simulation [12]. Each boundary layer is spatially defined with a constant refractive index, and the refractive index is assumed to remain steady between layers. This discretization enables the application of geometric ray tracing. The process begins by determining the intersection of a ray with the outermost boundary layer. Each boundary layer is represented as a mesh—a common technique for three-dimensional geometry representation—composed of points (vertices) and polygons (faces); in this work, the polygons are exclusively triangles. Once the intersection point between a ray and a polygon in the boundary layer is established, Snell’s law of refraction is applied to calculate the refracted ray, utilizing the angle of incidence relative to the polygon’s normal vector and the refractive indices on either side of the boundary. The refracted ray is then intersected with the subsequent boundary layer, and the refraction calculation is repeated. Building on these fundamental considerations, the following section presents the specific GPU-based simulation setup and explains how the IRIF data are integrated into our ray tracing approach.

## 3. Simulation Setup

**Figure 4:**
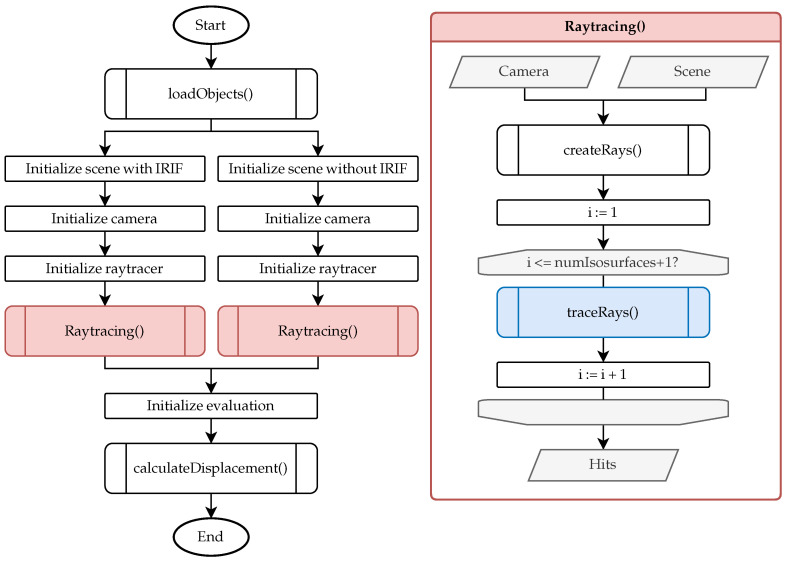
General procedure for calculating viewing ray deviations.

Figure 4 presents a flowchart outlining the comprehensive workflow of the ray tracing framework. The main program is depicted on the left, while the right side provides a detailed visualization of the ray tracing function.

Initially, the main program invokes the function *loadObjects*, which performs four fundamental tasks. At first, it decomposes a single structure containing multiple refractive indices (in *.vtu* format) obtained from the COMSOL simulation into individual boundary layers, each characterized by a unique refractive index (in *.vtm* format). Secondly, it converts these data from the *.vtm* format to the *.mat* format to ensure compatibility with the MATLAB-based (2024b) ray tracer. Thirdly, the function trims the boundary layers based on the fields of view of the optical components to reduce computational demands during the ray tracing process. Finally, it loads the measurement object into the simulation.

The scene, camera, and ray tracer are initialized twice using the boundary layers and the measurement object. The scene is set up both with and without the boundary layers to facilitate a comparison between refracted and unrefracted viewing rays in the final analysis. Ray tracing of the camera’s viewing rays through the scene is performed for each configuration. Unlike traditional ray tracing methods requiring explicitly defined light sources, our approach utilizes reverse (backward) ray tracing, meaning no external light source is modeled in the simulation. Instead, the hot-formed component acts as the light-emitting source due to its thermal radiation, which is directly modeled in the simulation. This is also illustrated in Figure 1, where no external illumination is present. Since rays are traced from the camera backward into the scene, this approach inherently accounts for the optical path reversibility principle and does not require defining a separate light source.

Initially, the camera viewing rays are generated. The optical camera center is defined at the coordinate origin. The sensor area is created at z=f focal length in *z*-direction. The sensor size in *x* and *y* is divided by the sensor resolution to obtain the center of each pixel corresponding to the camera ray. After normalization of the camera ray, it is transformed to the camera position and rotation. Subsequently, a loop is executed for tracing the viewing rays (*traceRays*) through the isosurfaces. The number of iterations equals the number of boundary layers in the scene plus one. In each iteration, the intersection points on the *i*-th boundary layer and the refraction angle of each ray are calculated. Since the intersection points of the rays from the last boundary layer to the measurement object are also determined, the total number of boundary layers is effectively increased by one. The output of the ray tracing function comprises the intersection points on the surface of the measurement object, which are subsequently utilized in the *computeDisplacement* function.

**Figure 5:**
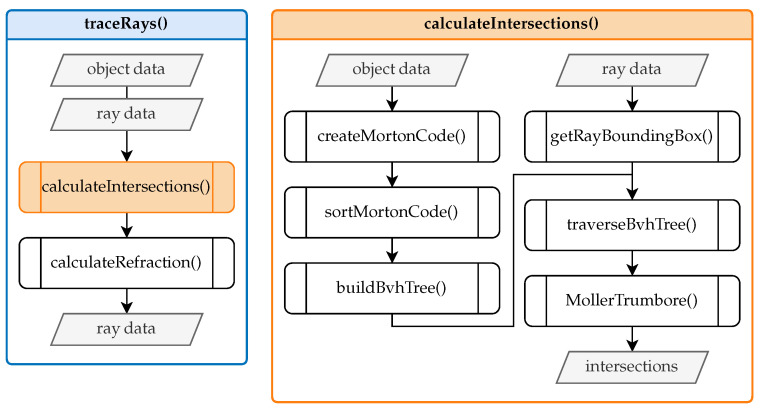
Core ray tracing functions.

The primary functions include tracing the rays and determining the intersection points. Figure 5 provides an overview of this process.

Given the input data, which include object and ray information, the *traceRays* function initially determines the intersections of the rays with objects by invoking the *calculateIntersections* function. If the intersected object is an interface with a specific refractive index, the *computeRefraction* function is subsequently executed. This function employs the surface normal vector and the incoming ray at the point of intersection to compute the refracted ray according to Snell’s law of refraction (refer to Figure 2). The refracted ray is then used for further ray tracing. If the ray reaches a measurement object, the tracing process terminates. The *calculateIntersections* function constitutes the core of the ray tracing process and is entirely based on the code provided by Leung [18]. This function is fully implemented in CUDA, enabling the calculations to be parallelized on the GPU. This approach significantly reduces runtime compared to CPU-based implementations.

Inside the *calculateIntersections* function, the *generateMortonCode* function creates a one-dimensional representation known as Morton code [19] for the centers of the three-dimensional triangles, capturing their spatial proximity. These Morton codes are sorted in ascending order using *sortMortonCode* to facilitate an efficient linear organization of the subsequent search tree. The *buildBvhTree* function then constructs a bounding volume hierarchy (BVH) [20] search tree using the sorted Morton codes to identify potential intersections between rays and objects efficiently. This process involves hierarchically enclosing the triangles within bounding volumes using bounding boxes. Afterward, each ray within a bounding box is encapsulated to traverse the BVH tree efficiently. This traversal (*traverseBvhTree*) checks for intersections between the ray envelopes and the BVH nodes, leading to the identification of potential collision candidates.

The calculation of the intersection points between the selected triangle of the isosurfaces and the camera’s viewing rays utilizes the Möller–Trumbore (*MollerTrumbore*) algorithm [21]. This algorithm, employing barycentric coordinates, is widely adopted for determining the intersection between a ray and a triangle in three-dimensional space. Having established the technical details of the GPU ray tracer and the discretization strategy, we now evaluate the method’s performance and accuracy under different conditions in Section 4.

## 4. Results

### 4.1. Preliminary Investigation

This subsection examines the characteristics of the developed ray tracing framework. Parameters that significantly affect the system’s performance are identified and analyzed. Investigating how the simulation responds to variations in these parameters seeks an optimal balance between computational runtime and result quality. Based on these analyses, suitable parameter configurations are determined. These preliminary studies also establish comparability with other ray tracing frameworks in the context of the ray tracer’s development.

#### 4.1.1. Parameter Selection

This section outlines the parameters that are varied in this chapter and provides the rationale for their selection. The parameters under consideration are as follows:

1. The number of viewing rays;
2. The number of boundary layers;
3. The number of polygons per boundary layer;
4. The smoothing of boundary layers;
5. The export configuration of the COMSOL simulation.

The number of viewing rays is a crucial parameter because increasing it leads to finer sampling and more detailed results, albeit at the cost of increased computational time. A quantitative analysis of the relationship between the number of viewing rays and computation time will be performed. Each camera pixel corresponds to a viewing ray, and the number of viewing rays varies according to the camera resolution.

It is hypothesized that increasing the number of boundary layers will enhance the quality of the results. The metric used to assess quality is described in Section 4.1.2. A proportional increase in computation time is anticipated due to the additional computational effort required.

The number of polygons per boundary layer influences the construction of the bounding volume hierarchy (BVH) tree, as introduced in Section 3, and the subsequent computation of intersections between viewing rays and polygons. An increase in this number is expected to lead to longer computation times, which will be quantitatively analyzed. A proportional relationship between the number of polygons and computation time is also assumed for the simulation of the IRIF. Additionally, it is assumed that the overall simulation accuracy improves with an increased number of polygons.

The mesh configuration in the COMSOL simulation affects the number of support points in the computational grid, which defines the resolution of the IRIF. The resolution of the iso-boundary layers during export can also be adjusted, affecting the number of exported polygons. The impact of these factors on computation time and ray tracing quality will be examined.

**Figure 6:**
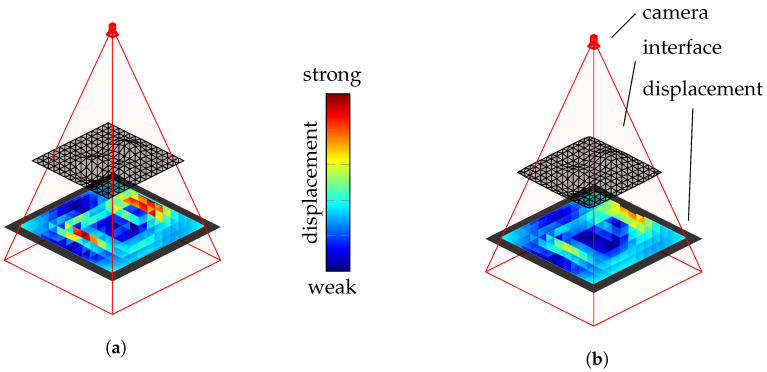
Reduction in edge formations using the Taubin filter: (**a**) the original and (**b**) with the applied Taubin filter.

The initial ray tracing results suggest that fewer polygons per boundary layer lead to increased edge formations in the viewing ray displacement. Furthermore, this section will investigate whether smoothing the coarse boundary surfaces enhances quality. Smoothing is performed using the Taubin filter, as presented in [22], which iteratively adjusts surface points to reduce irregularities and smooth the surface. The filter combines the Laplace operator and normal information to analyze and adjust local curvatures. Compared to other smoothing algorithms, such as mean filters or Laplace filters, the Taubin filter prevents surface shrinkage. Figure 6 illustrates smoothing using the Taubin filter.

The displacement of the camera’s viewing rays due to refraction at the boundary surface is color-coded on the object’s surface. The left side of Figure 6 displays the result without smoothing, while the right of Figure 6 shows the boundary surface after smoothing with the Taubin filter, which reduces the sharp edges in the viewing ray deviation. The impact of this smoothing on the quality will be discussed in Section 4.1.3.

A standard configuration of the ray tracer parameters is employed for all the investigations. The COMSOL simulation configurations defined in this work are listed in Table 1.

A cylinder with a length of 170 mm and a diameter of 27 mm is used as a test object. The refractive indices around the virtually 1100 °C hot cylinder are calculated to range from 1.00028 in the surroundings (at a temperature of 20 °C) to 1.00013 on the surface. COMSOL configuration C is utilized in the simulation as it represents an intermediate option in terms of the number of polygons. Thirteen boundary layers are imported into the ray tracing environment for each run and are reduced to the camera’s field of view.

The virtual camera has a sensor size of 6.7 mm × 5.6 mm. The camera resolution of 2448×2048 pixels is down-sampled by a factor of 0.5 in width and height, setting the total number of camera viewing rays to 1,253,376. This scaling is performed to limit the overall duration of the investigations to a manageable level. Furthermore, when using the boundary layers, the viewing rays are masked with the simulation results without boundary layers, so only those viewing rays that have previously intersected the object are considered.

The camera’s focal length is set to 10 mm, ensuring that the measurement object is fully captured in the image from the selected fixed camera position. The focal length of 10 mm is relatively wide-angle; however, based on our experience, it is still used in in-line quality control with cameras for visual inspection. The camera is positioned diagonally above the measurement object, with its optical axis directed at the center of the cylinder. The acceptable depth of field ranges from 50 mm to 250 mm; points outside this range are masked and not further considered.

The hardware utilized includes a Windows 10 PC with an AMD Ryzen 7 7700 8-core processor clocked at 3.8 GHz, 32 GB of RAM (4800 MHz), and an NVIDIA GeForce RTX 3060 with 12 GB of dedicated memory.

#### 4.1.2. Quality Metric

The reference for analyzing the number of boundary layers results from a simulation utilizing the maximum number of boundary layers, set to 20. This approach is based on the assumption that a higher number of boundary layers leads to more precise results. This is supported by studies conducted by Montagnino [8], who demonstrated that the accuracy of ray tracing improves with an increasing number of boundary layers. An extrapolation technique is applied to calculate the refractive index from point to point in an inhomogeneous refractive index medium. Using a constant refractive index gradient, ref. [8] calculated the error for various step sizes. A clear correlation was observed between smaller step sizes, which corresponded to more boundary layers and increased accuracy.

For the investigation of smoothing, the reference is the result of a simulation using a very fine-mesh boundary surface. The export configuration D, described in more detail in Table 1, is selected for this purpose. A coarse-mesh boundary surface from export configuration A is smoothed to test the smoothing effect. This approach allows for evaluating the impact of smoothing on the accuracy and quality of the simulation results.

**Figure 7:**
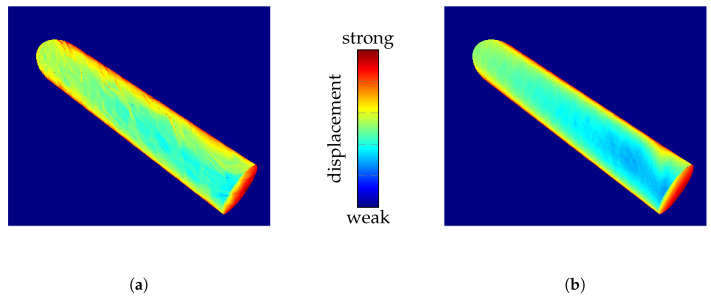
Illustration of the impact of COMSOL configuration on the simulation results. The color-coded viewing ray displacement on the surface of a cylinder during ray tracing through the IRIF is presented. (**a**) Configuration B+: normal mesh resolution and high isosurface resolution. (**b**) Configuration D: extra-fine mesh resolution and normal isosurface resolution.

To evaluate configurations within the COMSOL simulation, we have to select a configuration as a reference for the best quality. If polygon count alone were considered, configuration B+ would be preferred. However, Figure 7 illustrates more detailed results when using boundary surfaces from configuration D. This suggests that the resolution of the physical simulation, as opposed to merely the number of exported polygons, has a more significant impact on the quality of the simulation results. Therefore, configuration D is used as a reference configuration to quantify quality.

#### 4.1.3. Preliminary Results

**Figure 8:**
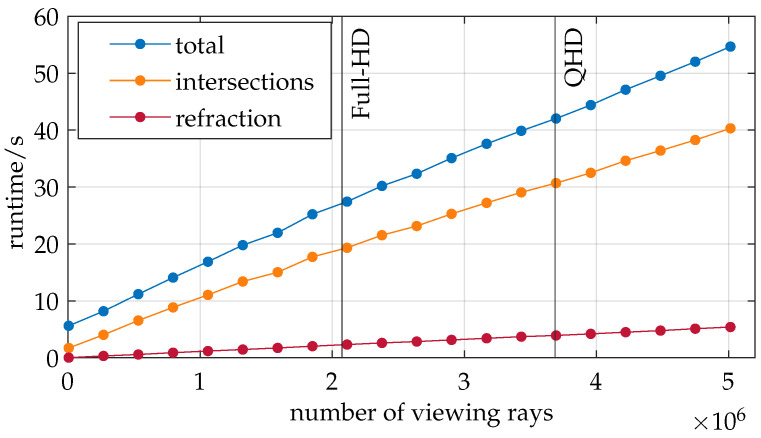
The number of viewing rays used in ray tracing exhibits a linear relationship with runtime.

The impact of various parameters of the developed ray tracer is further investigated with regard to runtime and the quality metrics defined in Section 4.1.2. Figure 8 depicts the runtime of the ray tracer versus the initial number of camera viewing rays.

In addition to the total runtime (blue), which includes all the processes from loading the boundary layers to calculating the viewing ray displacement, the program’s time spent finding intersections (orange) and calculating the refracted viewing rays (red) is also displayed. Vertical lines are provided for context, highlighting common camera resolutions. It should be noted that COMSOL configuration B is used instead of C in this analysis.

All three graphs exhibit a strongly linear trend. This behavior is anticipated for the intersection search since the Möller–Trumbore algorithm is executed for each viewing ray. Consequently, doubling the number of viewing rays results in a doubling of computational operations. The time complexity for the intersection search can thus be described as O(n). Similarly, the linear trend justifies a time complexity of O(n) for the refraction calculations as the viewing ray computations during refraction are vectorized.

This investigation confirms the system’s successful implementation. The trend in the total runtime suggests that the number of viewing rays does not significantly affect the runtime of other program components. Therefore, it is reasonable to state a time complexity of O(n) for the entire program relative to the number of viewing rays.

**Figure 9:**
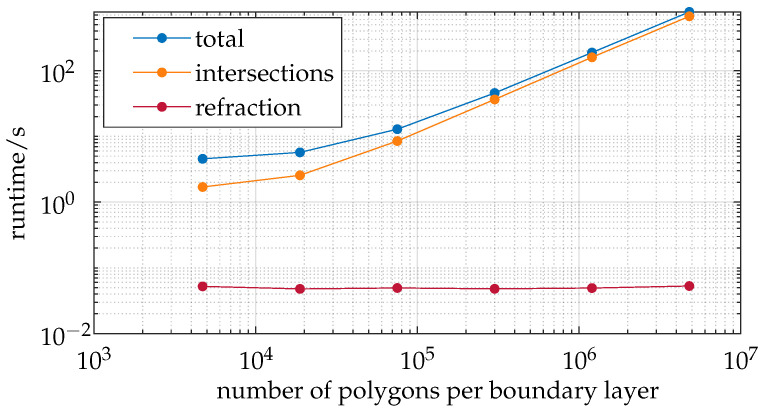
The number of polygons per boundary layer exhibits an approximately linear relationship with runtime.

Figure 9 analyzes the ray tracer with respect to the number of polygons input per boundary layer.

This analysis is based on a coarse mesh with an average of $4.7\times 10^{3}$ polygons per boundary layer. The times shown represent the cumulative runtimes of the respective processes, similar to the previous analysis. The polygons are quadrupled using the Midpoint-Split method, which is employed in rapid realistic computer visualization of 3D objects, as described in [23]. The number of polygons per boundary layer is logarithmically increased, reaching an average of $4.8\times 10^{6}$ polygons per boundary layer.

The analysis reveals that the refraction process exhibits constant behavior, indicating that it is independent of the number of polygons per boundary layer (O(1)). This outcome is expected since this process only utilizes the normal vectors of the intersected polygons, which are determined by the viewing rays that remained constant in this series of measurements. The duration of intersection searching proves to be highly dependent on the number of polygons per boundary layer and is the dominant factor in the total runtime. Both the total runtime and the intersection search time exhibit approximately linear growth. However, the graphs appear to show a disproportionately steep increase at the beginning, up to approximately $10^{5}$ polygons per boundary layer. Considering the logarithmic scale, this deviation should be interpreted with caution and should not be overemphasized.

The time complexities indicate that both the creation and traversal of the BVH search tree and the execution of the Möller–Trumbore algorithm can be regarded as having a time complexity of O(n). This is because doubling the number of polygons while keeping the number of rays constant results in doubling algorithmic calls. The creation of the BVH search tree is also noted by Apetrei [24] as having a complexity of O(n). It is assumed that the duration of other program components, such as the refraction calculation, is minimally or not significantly affected by the number of polygons. This explains the increasing proportion of intersection search time in the total computation time (from 37.3% to 85.8%).

**Figure 10:**
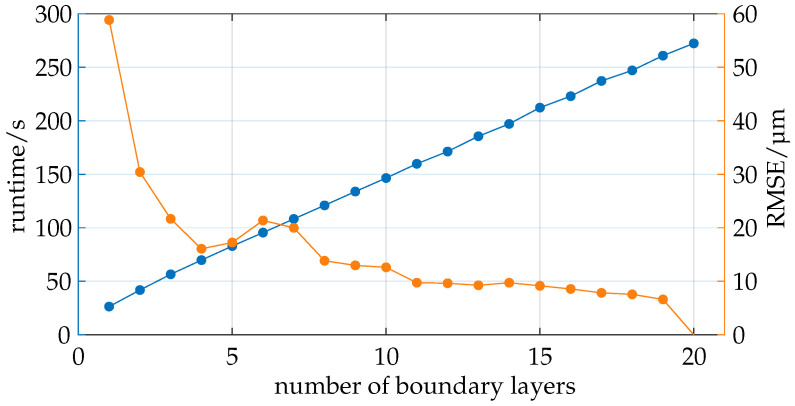
The number of boundary layers used exhibits a linear relationship with runtime and a diminishing trend with respect to the quality metric.

In conclusion, the intersection search and the total computation time can be considered to have a time complexity of O(n) concerning the number of polygons. The study illustrated in Figure 10 examines the effect of the number of boundary layers on the runtime and quality of the developed ray tracer.

Ray tracing is performed using exports from the COMSOL simulation with varying numbers of boundary layers, ranging from 1 to 20. The results are evaluated based on two primary metrics: the Root Mean Squared Error (RMSE) in micrometers, which indicates the relative quality of the simulation compared to the simulation with 20 boundary layers, and the total runtime in seconds, encompassing all the process steps, including loading, preprocessing, simulation, evaluation, visualization, and storage.

It can be observed that the total runtime increases linearly with the number of boundary layers, which is expected since the boundary layers influence various aspects of the program, including loading, preprocessing, ray tracing, visualization, and storage. Notably, the intersection calculation dominates the total process duration, with its share increasing from 81.9% with one boundary layer to 90.8% with twenty boundary layers.

It is noticeable that the quality, as measured by the RMSE, does not follow a linear trend. A significant improvement in quality is observed when increasing from one to two boundary layers, followed by a slower improvement. A local maximum in RMSE is reached with six boundary layers, after which the quality improves again. The increase in quality between 11 and 19 boundary layers is relatively minor.

The results suggest that a minimum of 11 boundary layers should be used as the quality improves noticeably up to this point and the computational costs are linearly proportional. For this scenario, using 13 boundary layers seems to be advisable to ensure a margin of safety that can account for potential further influences on quality, such as changes in intrinsic and extrinsic camera parameters.

**Figure 11:**
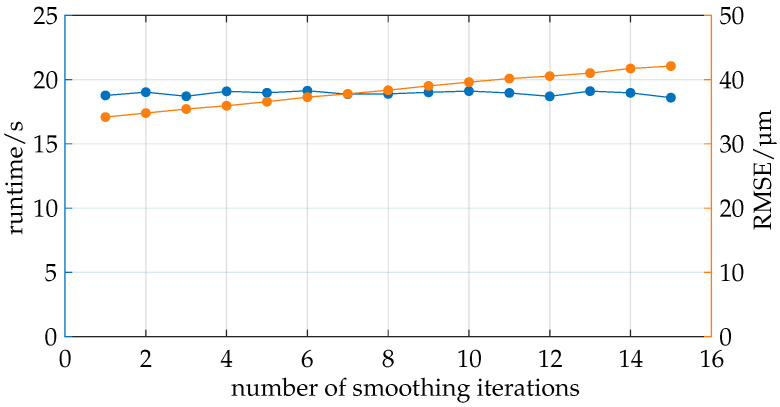
Smoothing the boundary layers has no impact on runtime. The RMSE relative to the reference increases linearly with the number of smoothing iterations.

The study illustrated in Figure 11 aims to analyze the impact of smoothing the boundary layers using a Taubin filter.

The number of iterations is varied while the scaling factor remains constant at 0.20–0.21. A relatively low scaling factor is chosen because higher scaling values led to errors in ray tracing due to the smoothing of the boundary layers. Smoothing caused larger boundary layers to intrude into the volume of smaller boundary layers, altering the order of the layers, which in turn led to errors in ray tracing.

As in the previous analysis, the total runtime and RMSE are recorded. The runtime remained nearly constant, as expected, since the number of polygons did not change; only the positions of the vertices did. The increasing number of smoothing iterations proved to be negligible in the total computation time.

The RMSE is calculated compared to simulations with significantly finer boundary layers (more polygons per boundary layer). The boundary layers used here are relatively coarse and tend to form edges in the results, as explained in Section 4.1.1. The RMSE shows a slight linear increase, disproving the hypothesis that smoothing the meshes would approximate the results of finer meshes. The quality decreases with increasing smoothing.

It should be noted that a general conclusion cannot be drawn here as the geometry of the boundary layers can change entirely due to the geometry of the measurement object, and the smoothing parameter space is not sufficiently explored in this study. Due to the unpromising results of this study, no further investigation of smoothing is conducted.

**Figure 12:**
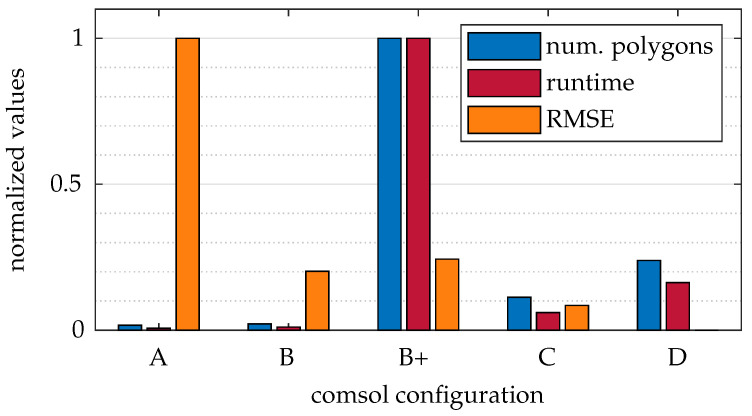
Runtime and RMSE values for different simulation-based mesh configurations.

Figure 12 presents a comparison of the various COMSOL configurations, which include both the resolution of the refractive index field simulation (A, B, C, or D) and the number of exported polygons (+). A detailed description of these configurations can be found in Table 1 and Section 4.1.1. The figure displays the runtime of a ray tracing run, the RMSE relative to the D configuration, and the number of polygons. The values are normalized to their respective maximum values.

Excluding B+, an inverse correlation of runtime to RMSE is observed, which aligns with the expectations. Therefore, a trade-off must occur between runtime and quality. Ignoring D, configuration B shows the best RMSE in relation to runtime. It should be noted that, for configurations A and B, we had to eliminate the three isosurfaces closest to the cylinder because they clipped into the cylinder. This breaks the hierarchical ray tracing order and results in faulty simulation results.

Configuration B+ proves that the isosurface setting has no influence on the quality of the simulation but does have an immense effect on the runtime. Although the resolution of the isosurfaces improves, the resolution of the physical simulation remains the same. The information content between B and B+ is identical. Accordingly, the output configuration of the isosurfaces should not be increased. Configurations A and B+ are unsuitable for applications for quality and runtime reasons, respectively.

It is also important to note that these results do not provide information about the runtime or quality of the simulation within COMSOL itself. However, practical experience has shown that higher resolutions in the COMSOL simulation (configurations A, B, C, and D) increase runtime. The simulation quality directly depends on the resolution, improving progressively from A to D. These preliminary findings clarify the computational constraints and inform our further exploration of light deflection in inhomogeneous refractive index fields, as examined next.

### 4.2. Light Deflection on Optical Systems

In this section, we characterize the IRIF’s influence on the displacement of viewing rays within a camera system. The primary objective is to determine an optimal position for an optical measurement system that minimizes errors in comprehensive surface measurements. Additionally, by deepening our understanding of the IRIF-induced displacements in viewing rays, we assess the necessity of employing the developed ray tracing method.

#### 4.2.1. Parameter Setup

The settings of the developed ray tracer are examined to optimize it for high-quality results with minimal computational time. Initially, the focus is on characterizing the ray tracer and comparing it with existing solutions. The current analysis, however, shifts to characterizing the displacements in viewing rays induced by the IRIF with respect to the extrinsic camera parameters. This analysis is based on the same COMSOL simulation of an IRIF surrounding a uniformly heated cylinder of Section 4.1.2. Based on the insights from Section 4.1.3, we have selected the ray tracing configuration outlined in Table 2.

The extrinsic camera parameters specify the camera’s position and orientation. The camera’s position is varied to sample the area around the cylinder while remaining outside the IRIF. To facilitate this, an ellipsoid is defined around the measurement object. The camera’s distance is selected so that it is positioned outside the largest boundary layer at every location. The semi-axis ratio of the ellipsoid is derived from the cylinder’s dimensions (length to diameter). The camera’s optical axis is always directed toward the center of the cylinder, which corresponds to the origin of the coordinate system. The *x*-axis of the camera’s coordinate system always lies within the xy-plane of the world coordinate system. With the constraint that the *y*-axis of the camera coordinate system always points in the positive *z*-axis direction of the world coordinate system, the orientation is mathematically well defined.

The investigations are confined to a 90° segment of the elliptical orbits, under the assumption that the results are axisymmetric with respect to the *x*- and *y*-axes. This assumption stems from the cylinder’s axisymmetry. However, there is no symmetry with respect to the *z*-axis because the refractive index field propagates opposite to the direction of gravity, which points in the negative *z*-direction in this context.

#### 4.2.2. Investigation Metrics

To quantitatively compare the extrinsic camera parameters, the following metrics are employed:

1. The mean Euclidean distance between the IRIF-refracted and IRIF-unrefracted lines of viewing: $\bar{d}$;
2. The mean lateral and axial resolutions: $R_{\mathrm{lat}}$ und $R_{\mathrm{ax}}$;
3. The mean angle of incidence between the line of sight and the surface: $\theta_{\mathrm{inc}}$;
4. The computation time of the ray tracing simulation: $T_{\mathrm{sim}}$;
5. The coverage of the measurement object’s surface by the lines of sight: $\eta_{\mathrm{surf}}$.

The primary metric for assessing the quality of the surface representation of the measurement object is the Euclidean distance of the line-of-sight deviation caused by the inhomogeneous refractive index field. For each line of sight that intersects the object’s surface, a displacement (*d*) is computed. Owing to the large amount of data, only the mean value of all the displacements for a given camera position and orientation is considered ($\bar{d}$).

In addition to the magnitude of the light deflection effect, the lateral and axial resolutions are crucial metrics for determining the quality of measurements using optical sensors. Low resolution in either direction leads to sparse sampling of the measurement object’s surface. Axial resolution refers to the minimal local point spacing along the camera’s optical axis.

**Figure 13:**
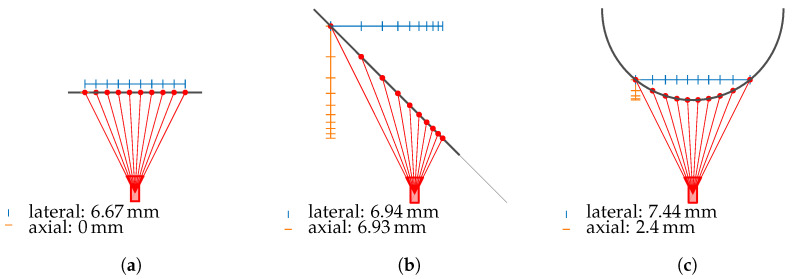
Resolutions depending on the angle of incidence with (**a**) resolutions at $\theta_{\mathrm{inc}}=0{}^{\circ}$; (**b**) describes the resolutions at $\theta_{\mathrm{inc}}=45{}^{\circ}$, and (**c**) visualizes resolutions for a cylinder.

The point cloud of the line-of-sight intersection points with the measurement object’s surface is transformed into the camera coordinate system. In this system, the optical axis of the camera points in the positive *z*-direction, with the camera located at the origin. The Euclidean distance in the *z*-direction to the nearest neighboring point, identified using the K-Nearest Neighbor (KNN) algorithm, represents the axial resolution. The Euclidean distance to the nearest neighboring point within the xy-plane defines the lateral resolution. As illustrated in Figure 13, the axial and lateral resolutions, in addition to the intrinsic camera parameters, also depend on the relative position of the camera to the measurement object’s surface. Therefore, it is essential to include these resolutions as metrics in the analysis.

Given the findings from the previously presented metrics, it is hypothesized that the angle of incidence of the line of sight upon the measurement object’s surface plays a significant role. This angle is calculated by determining the angle between the direction vector of the incident line of sight and the normal vector of the intersected surface polygon. For each camera position, the mean of all the angles is computed.

Furthermore, the surface coverage of the measurement object from a specific camera position is examined. High coverage reduces the number of measurements required for a complete geometric capture of the surface, thereby decreasing the measurement time and effort. The total surface area is calculated as the sum of the areas of all the polygons on the cylinder. Coverage is defined as the proportion of the intersected polygons relative to the total surface area of the cylinder.

Additionally, the total computation time of the ray tracing process from each camera position is analyzed. This process includes loading the boundary layers and the cylinder, preprocessing, ray tracing, evaluation, visualization, and storage. Minimizing the computation time is important to expedite the search for error-minimizing measurement poses.

#### 4.2.3. Light Deflection Results

**Figure 14:**
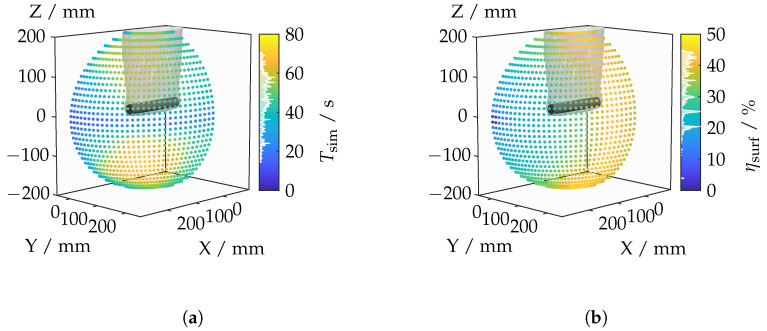
Calculation time (**a**) and measurement object coverage (**b**) for each camera position.

This section presents and discusses the results of the light deflection investigations. The results are displayed as a point cloud in three-dimensional space, with the color of the points encoding the corresponding values for the selected camera positions. The analysis is conducted from a perspective outside the elliptical orbits. Additionally, both the cylinder and the outermost boundary layer of the IRIF are faintly indicated. The discussion begins with the computation time depicted in Figure 14a. The computation time spans a broad range from 14.2s–70.9s and depends on the camera’s position.

**Figure A1:**
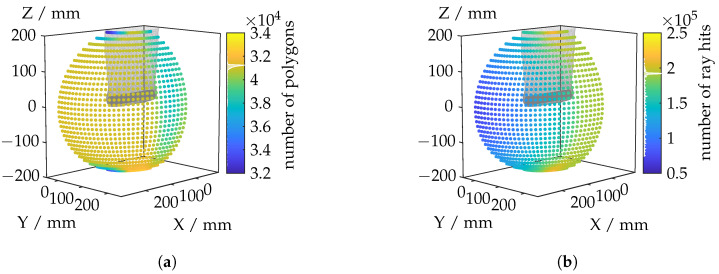
Number of polygons (**a**) and number of ray hits (**b**) per camera position.

The variation in the duration of the intersection search may be attributed to differences in the number of polygons and the intersected lines of sight. One hypothesis suggests that the number of intersected lines of sight is critical as only those that initially intersect the cylinder without the refractive index field are used for ray tracing through the refractive index field. However, there is no direct correlation with the number of intersected lines of sight (see Figure A1b).

Similarly, the number of polygons used for ray tracing after the boundary layers have been intersected by the camera’s field of view is not decisive; this parameter also shows no correlation with computation time (see Figure A1a). It is hypothesized that the time required for building or traversing the BVH search tree may vary depending on the camera’s position. Given that computation time does not have immediate relevance to measurement planning, it will not be further elaborated upon in this context.

The analysis of object coverage in Figure 14b reveals varying results depending on the viewing angle. When observed along the lateral surface, a significantly higher object coverage is evident, reaching a maximum of 44.9%. In contrast, a frontal view of the top surface results in a low object coverage of only 3.7%. These differences can be explained by the varying contributions of different parts of the object’s surface, with the lateral surface contributing a much larger portion to the total surface area compared to the top surface.

A notable transition occurs with increased rotation of the camera, where the object coverage suddenly increases. This is due to the enhanced visibility of the lateral surface, which was previously limited by the depth of field. For measurement planning, this indicates that both lateral and frontal measurements are necessary to capture the full geometry of the object. Lateral measurements require rotations around the cylinder to ensure comprehensive coverage, while frontal measurements should be conducted at both ends of the cylinder.

**Figure 15:**
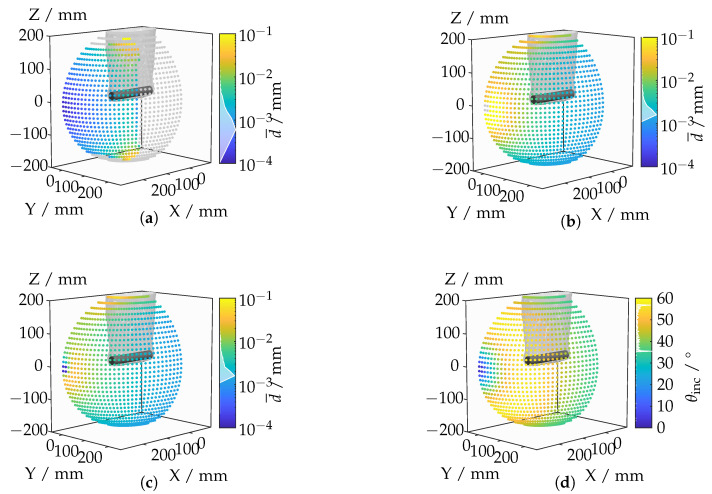
(**a**) Displacements for the surface area of the cylinder head. (**b**) Displacements for the lateral surface area of the cylinder. (**c**) Displacements for the total surface area of the cylinder. (**d**) Angle of incidence.

Figure 15 illustrates the mean displacement of the lines of sight caused by refraction at the boundary layers of an inhomogeneous refractive index field. Figure 15c encompasses the average deviations of all the lines of sight. A lateral view results in comparatively small displacements, whereas rotation toward the top surface of the cylinder leads to increasing mean displacements. Positions where only the top surface is visible exhibit minimal deviations.

A specific analysis of the lines of sight that intersect the lateral surface of the cylinder, shown in Figure 15b, reveals a similar distribution to that of the entire cylinder surface. However, particularly strong deviations occur when rotating toward the top surface of the cylinder, which is attributable to the shallower angle of incidence on the lateral surface. This shallower angle causes a refraction-induced change in angle to have a significantly larger effect.

The examination of only those lines of sight that intersect the top surface of the cylinder, as shown in Figure 15a, generally indicates lower displacements. This can be explained by the alignment of the camera’s optical axis with the normal vector of the flat top surface, resulting in less pronounced displacements. The analysis suggests that the angle between the optical axis and the surface is critical. Even a few outliers can lead to significant displacements due to the averaging process. There is a correlation between the angles of incidence of the lines of sight and the optical ray displacement. A diagonally oriented viewing angle of the camera is associated with larger angles of incidence. Frontal viewing results in the smallest angles of incidence, while lateral viewing, despite having relatively low angles of incidence, still exhibits higher values due to the curved surface compared to frontal viewing.

The discrepancy between angles of incidence and optical ray displacement in the immediate vicinity of the frontal view can be explained by the impact of outliers on the mean calculation. The displacement of the lines of sight on the lateral surface can be several orders of magnitude greater than in the direct vicinity of the frontal view. However, the angle can only increase by a maximum of 90°, which represents a relatively small magnitude. Consequently, the change in angle has a less significant impact on the mean value.

**Figure 16:**
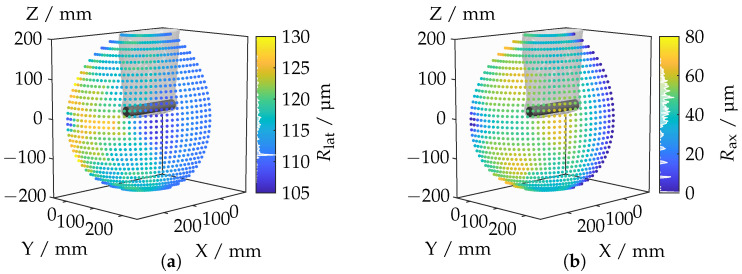
Lateral (**a**) and axial (**b**) resolutions for each camera position.

Figure 16 depicts the mean lateral and axial resolutions. It is important to note that higher resolution values indicate poorer quality as they represent the Euclidean point distance either along the optical axis (axial) or perpendicular to the optical axis (lateral) of the camera. The mean lateral resolution deteriorates, particularly when the camera is rotated toward the top surface of the cylinder (see Figure 16b). This deterioration can be attributed to the fact that, as the angle to the surface increases, the distance to the camera tends to become larger. Consequently, the lines of sight spread out more, leading to degraded lateral resolution. Even at its minimum, the lateral resolution remains significantly above zero as the distance between the camera and the object never falls below a certain threshold. In contrast, an axial resolution of zero is possible, as evident in the schematic diagram in Figure 13a.

A significant jump in lateral resolution occurs when the proportion of the top surface decreases. A similar deterioration in axial resolution is observed for camera positions oriented toward the top surface, with specific point areas being particularly affected. These areas are characterized by a distinctly diagonal perspective. Interestingly, areas with poor axial resolutions tend to have better lateral resolutions. It is worth noting that direct frontal and lateral perspectives on the top and lateral surfaces, respectively, offer very good lateral and axial resolutions, which can be attributed to the nearly frontal angle of incidence. With a better understanding of how IRIFs impact basic optical configurations, we extend the analysis to complex application-relevant geometries in Section 4.3.

### 4.3. Complex Components

In this subsection, the ray tracing framework is applied to more complex geometries and varying refractive index distributions. The components under investigation are a hybrid tailored forming cylinder, a bevel gear, and a wishbone. According to [12], these components exhibit significantly different IRIF distributions, presenting unique challenges for optical measurements.

#### 4.3.1. Hybrid Cylinder

The tailored forming component combines a nickel-based alloy (W. Nr. 2.4602, also known as Hastelloy C-22/UNS N06022) [25] and an aluminum alloy (W. Nr. 3.2315, commonly EN AW-6082/UNS A96082) [26] with a gradient-based temperature profile, resulting in a non-uniform IRIF around the component.

**Figure 17:**
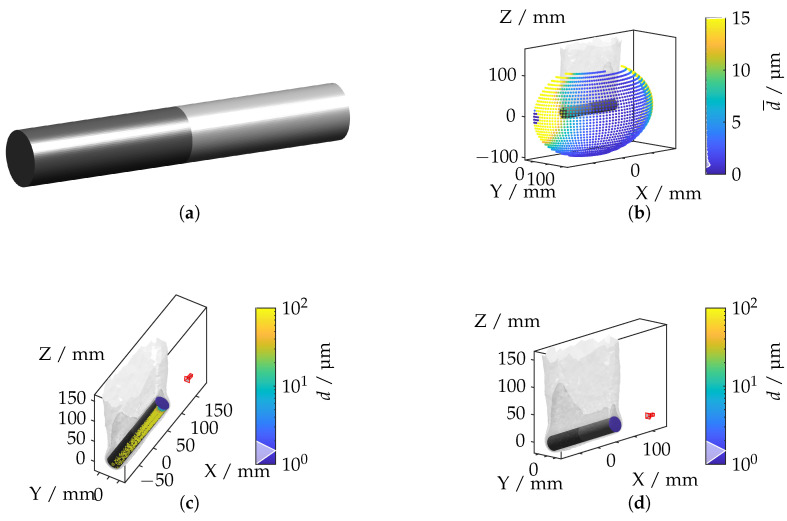
(**a**) Steel–aluminum hybrid cylinder geometry. (**b**) Mean displacement for each camera position. (**c**) Camera position with the highest displacement. (**d**) Camera position with the lowest displacement.

Figure 17a illustrates the geometry of the hybrid tailored forming cylinder. The spatial distribution of the mean displacement, depicted in Figure 17b, highlights that the highest displacements occur between the transition area between the cylinder head and the cylinder surface. The camera positions corresponding to the maximum and minimum displacements are shown in Figure 17c,d, respectively. The highest displacement values are observed when the camera is oriented along the material interface, where light rays experience maximum refraction. Conversely, minimal deviations occur when the camera is axially aligned with the cylinder head as light propagation in this configuration is less affected by the IRIF.

#### 4.3.2. Bevel Gear

**Figure 18:**
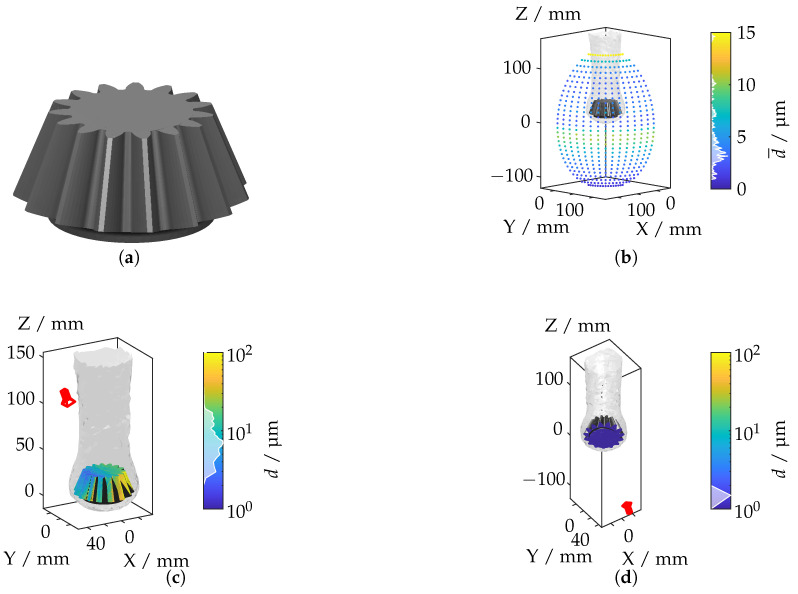
(**a**) Bevel gear geometry. (**b**) Mean displacement for each camera position. (**c**) Camera position with the highest displacement. (**d**) Camera position with the lowest displacement.

The geometry of the bevel gear, characterized by its conical teeth, is presented in Figure 18a. Non-uniform heating during the thermal process induces an asymmetric IRIF, particularly pronounced around the gear teeth.

The mean displacement distribution, shown in Figure 18b, reveals significant light deflections in regions with steep teeth gradients and high-temperature variations. Figure 18c,d depict the camera positions corresponding to the maximum and minimum displacement values, respectively. The largest displacements are observed when the camera is positioned to capture the gear teeth at oblique angles, where the refractive index gradients are most intense. In contrast, minimal displacements occur when the camera is aligned along the symmetry axis of the gear, resulting in reduced light deflection due to the relatively uniform IRIF.

#### 4.3.3. Wishbone

**Figure 19:**
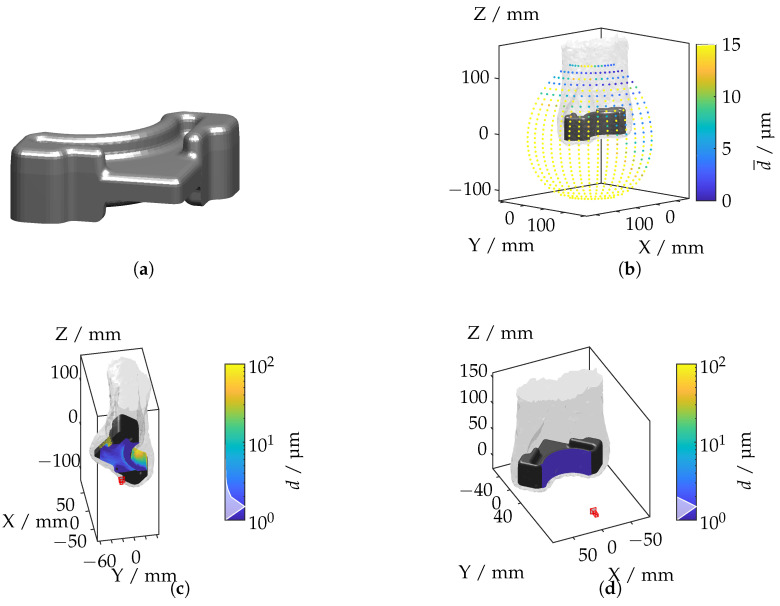
(**a**) Wishbone geometry. (**b**) Mean displacement for each camera position. (**c**) Camera position with the highest displacement. (**d**) Camera position with the lowest displacement.

The wishbone component, depicted in Figure 19a, features a complex geometry with curved surfaces and variable cross-sections.

The IRIF distribution, visualized through the mean displacement in Figure 19b, indicates that the highest light deflections occur in areas of pronounced curvature, reflecting the impact of non-uniform refractive index variations. Figure 19c,d illustrate the camera positions corresponding to the maximum and minimum displacement values. The highest displacements are observed when the camera is aimed at regions with significant curvature, where the IRIF gradients are strongest. Minimal displacements are noted for axial alignments, where the camera views the component’s central axis, reducing the effects of refractive index variations. The next subsection shifts the focus to a specific feature, the bevel gear tooth, to evaluate the methodology at a finer level of detail.

### 4.4. Feature Analysis: Bevel Gear Tooth

This section focuses on applying the ray tracing framework to a specific feature: a single tooth of a bevel gear. The objective is to assess the framework’s ability to calculate displacements caused by the IRIF while simultaneously evaluating key metrics, including coverage, mean axial resolution, mean lateral resolution, and a combined metric. Unlike prior analyses that centered on identifying poses with minimal overall displacement, this investigation emphasizes optimizing a pose using a multi-criteria combined metric.

#### 4.4.1. Metrics and Methodology

To ensure a comprehensive evaluation, the combined metric integrates four distinct criteria: displacement, coverage, mean axial resolution, and mean lateral resolution. The computation follows a two-step process:

1. **Normalization and Directional Alignment of Metric Distributions:**
  Each metric distribution is normalized over the range defined by its minimum value $m_{P0}$ and the 95th percentile value $m_{P95}$. This normalization maps all metrics to the interval [0,1], facilitating comparability. Additionally, the coverage metric is inverted to ensure that minimizing all metrics results in an optimized solution. The normalized metric for each $m_{i}\in M$ is computed as
  (2) $$m_{\mathrm{norm},i}=\frac{m_{i}-m_{P0}}{m_{P95}-m_{P0}},\;M_{\mathrm{norm}}=\left\{m_{\mathrm{norm},i}\in \left[0,1\right]\right\}.$$
2. **Weighted Aggregation of Metrics:**
  A weighted sum combines the normalized metrics into a single composite metric:
  (3) $$M_{\mathrm{combined}}=w_{d}\;\cdot \;{\bar{d}}_{\mathrm{norm}}+w_{\eta }\;\cdot \;\left(1-\eta_{\mathrm{surf},\mathrm{norm}}\right)+w_{\mathrm{ax}}\;\cdot \;R_{\mathrm{ax},\mathrm{norm}}+w_{\mathrm{lat}}\;\cdot \;R_{\mathrm{lat},\mathrm{norm}},$$
  where the weights reflect the relative proportion of each metric:
  (4) $$w_{d}=0.5,\;w_{\eta }=0.2,\;w_{\mathrm{ax}}=0.15,\;w_{\mathrm{lat}}=0.15.$$

**Figure A2:**
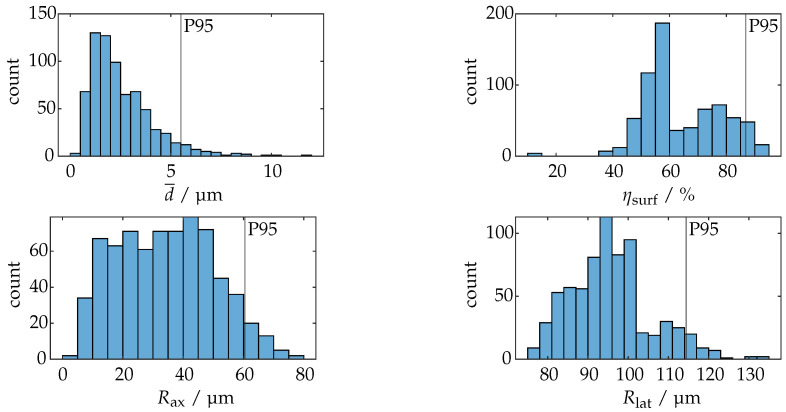
Distributions of the four metrics for the simulation from Figure 20.

**Figure A3:**
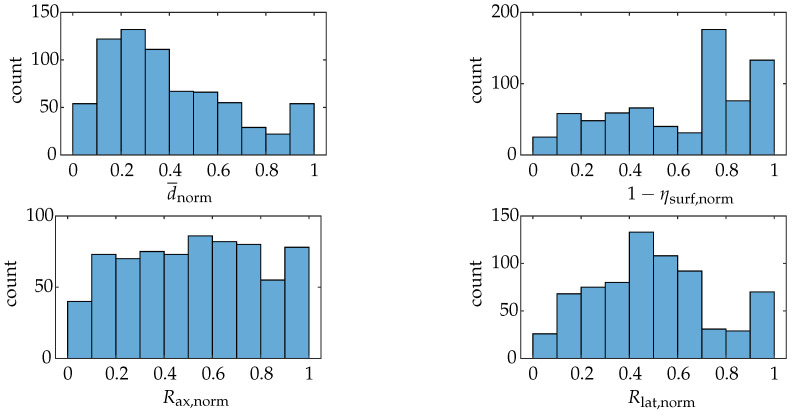
Normalized distributions of the four metrics for the simulation from Figure 20.

This approach ensures balanced optimization by accommodating both displacement minimization and broader measurement criteria. The weights were based on expert knowledge but can be adjusted according to the measurement scenario. The calculation of the combined metric is illustrated in the histograms shown in Figure A2 and Figure A3.

#### 4.4.2. Results and Interpretation

**Figure 20:**
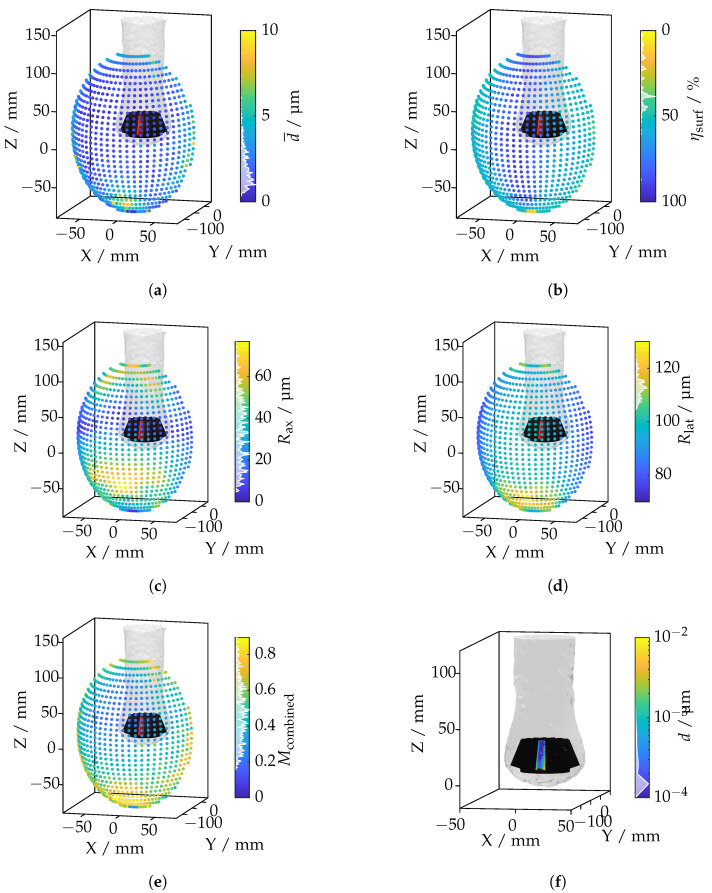
Investigation of a bevel gear tooth based on ray tracing metrics: (**a**) total mean displacement per camera position; (**b**) total coverage per camera position; (**c**) total mean axial resolution per camera position; (**d**) total mean lateral resolution per camera position; (**e**) combined metric per camera position; (**f**) camera position with the lowest displacement value.

The results are illustrated in Figure 20, showing the spatial distributions of the evaluated metrics across different camera positions.

Figure 20a illustrates the total mean displacement across camera positions. Maximum displacements occur at oblique angles, where the IRIF causes significant light deflection around the tooth. In contrast, minimal displacement is observed at frontal angles, where the IRIF gradient is less effective. The coverage metric in Figure 20b highlights regions where the camera captures the entire tooth surface. Low-coverage positions are generally associated with oblique viewing angles that restrict the field of view. Figure 20c,d display the axial and lateral resolutions, respectively. The axial resolution decreases when the optical axis is nearly parallel to the tooth surface, resulting in large point spacings in the axial direction. The lateral resolution worsens at extreme viewing angles due to sparse sampling perpendicular to the optical axis. The composite metric, shown in Figure 20e, integrates the individual metrics with their respective weights. This metric effectively identifies camera positions that balance displacement minimization, sufficient coverage, and acceptable resolutions. The camera position with the lowest combined metric is shown in Figure 20f. This position represents an optimal configuration that balances displacement minimization with other critical criteria, ensuring an efficient and effective measurement pose. Having assessed both broad and feature-level scenarios, we now synthesize and discuss the broader implications of these findings in Section 5.

## 5. Discussion

This study introduces a GPU-accelerated ray tracing framework to simulate the effects of IRIFs on optical measurements in hot-forming processes. By leveraging existing IRIF data from COMSOL Multiphysics simulations, the framework efficiently calculates the deflection of light rays caused by temperature-induced density fluctuations in the surrounding air. The results provide valuable insights into the magnitude and spatial distribution of measurement errors due to IRIFs, enabling the optimization of measurement positions to enhance the accuracy of optical geometry reconstruction systems.

A primary simplification inherent in the approach is the reliance on geometric optics for ray tracing. This decision is justified by the specific application requirements, which involve designing and positioning planar three-dimensional optical sensors that necessitate computational runtimes in the order of seconds to minutes. Alternative methods, such as the numerical solution of differential equations for the ray path, are computationally intensive and unsuitable for inline applications requiring rapid computations. In this context, the IRIF simulated using COMSOL is discretized into iso-index boundary layers to enable efficient ray tracing. It is assumed that, with sufficiently fine discretization, the simulated ray paths closely approximate the real ray paths, as analyzed in Section 4.1.3.

**Figure 21:**
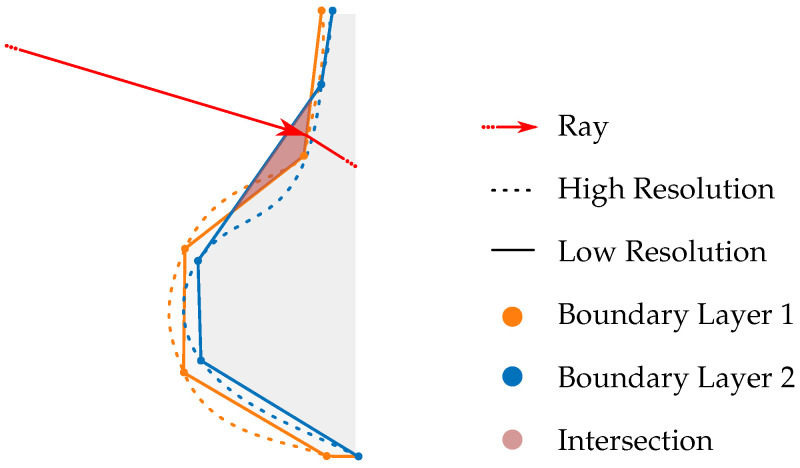
The intersection of boundary layers can lead to ray tracing errors as the boundary layers are sequentially incorporated into the ray tracing process hierarchically.

Another assumption is that the boundary layers are arranged in descending order of refractive index. Due to the high temperature of the observed object, the air layer closest to the surface is the hottest and, therefore, has the lowest density and refractive index. Ambient air, being cooler, features a higher refractive index. This arrangement enables considering only one boundary layer per ray tracing iteration, reducing the number of polygons and decreasing the computational runtime. However, a notable drawback arises when the number of boundary layers increases significantly. Overlaps of boundary layers due to their limited representation as triangles can lead to errors in the ray tracing process, as visualized in Figure 21.

These overlaps, particularly under conditions of low resolution and close spatial proximity of the boundary layers, result in inner boundary layers intersecting with outer ones. Consequently, the viewing ray may not reach the inner boundary layer after refracting at the outer layer. Instead, the ray may encounter the inner side of the following boundary layer due to the closed geometry and sequential consideration of the layers during the ray tracing iteration. By filtering out viewing rays that intersect with the inner side of a boundary layer, errors in the evaluation process can be avoided. However, this exclusion introduces certain limitations, potentially affecting the comprehensiveness of the simulation.

Simplifications are also present in the camera model, where pinhole camera models without distortion are utilized. The depth of field is binarized and presented as an allowable interval of ray lengths, with viewing rays always passing through the pixel center. These simplifications are acceptable for quantifying light deflection effects but may not fully represent the complexities of real optical systems. Implementing a more comprehensive camera model is recommended for the precise quantification of measurement errors in optical sensors.

The versatility of our approach is demonstrated by its successful application to complex geometries such as the bevel gear and wishbone. These geometries introduce unique challenges due to their irregular shapes and varying IRIF distributions. Since our method is based on a triangulated representation of surfaces, it is inherently adaptable to arbitrarily shaped components. Our results show that light deflection effects can be effectively analyzed for components with steep gradients and discontinuities in their refractive index fields. This supports the generalization of our methodology to a wide range of industrial applications. While further refinements could improve the accuracy for highly concave or internally reflective surfaces, our current results confirm that the method is not limited to specific component types.

The findings reveal that significant light deflections occur in regions with steep IRIF gradients, typically near material interfaces or geometrical discontinuities. The ability of the framework to accurately simulate these effects highlights its potential for improving the accuracy of optical measurements and optimizing measurement planning for industrial components. These results align with prior research [12], underscoring the critical role of IRIF-induced light deflections in precise measurement processes. The ray tracing framework is essential for addressing the complexities of measuring components with heterogeneous materials and intricate geometries, enabling more reliable calibration and correction techniques.

In the feature analysis of the bevel gear tooth, the framework demonstrated its capability to calculate displacements and evaluate multi-criteria metrics for specific features accurately. The introduction of the combined metric provides a structured methodology for optimizing camera poses, accounting for trade-offs between displacement, coverage, and resolution. This multi-criteria approach enhances the framework’s applicability to complex measurement scenarios, where a singular focus on displacement minimization may overlook practical measurement constraints. The methodology underscores the importance of integrating diverse metrics to achieve comprehensive measurement planning for intricate geometries.

### Limitations of the Method

Despite providing valuable initial insights, several further limitations of the presented simulation approach must be acknowledged. First, the model relies exclusively on geometrical optics (Snell’s law), omitting wave-optical phenomena such as diffraction and interference. Second, the method assumes that only a single light ray reaches each camera pixel, neglecting the potential effects of subsurface scattering or multiple surface reflections. Moreover, the air surrounding the hot component is treated as a static, layer-wise homogeneous refractive index field, ignoring turbulent or random density fluctuations that could introduce additional time-varying light deflections. A simple pinhole camera model was employed, excluding real-world lens distortions and chromatic aberrations. While this approach yields a practical first approximation of the refractive effects caused by an inhomogeneous refractive index field, more advanced optical or fluid-dynamics models would be required for highly complex or turbulent regimes to achieve a more precise experimentally validated representation. To conclude, the following section summarizes the key takeaways from this work and outlines potential avenues for future research.

## 6. Conclusions

This research presented a GPU-accelerated ray tracing framework to simulate light deflection in inhomogeneous refractive index fields around hot-forming components. Utilizing IRIF data from COMSOL Multiphysics simulations, the framework effectively computes the viewing ray displacements caused by temperature-induced air density fluctuations. The simulations highlight the significant impact of IRIFs on optical reconstruction, particularly in scenarios involving complex geometries and uneven temperature distributions.

The findings demonstrate that optimizing the measurement positions can substantially mitigate the measurement errors induced by IRIFs. The framework enhances the accuracy of optical geometry reconstruction in hot-forming applications by identifying camera positions that minimize light deflection while ensuring adequate coverage and resolution. The scalability and computational efficiency of the GPU-accelerated approach make it a viable tool for industrial applications where time and resources are critical.

In summary, the developed framework advances the understanding of IRIF effects on optical measurements. It provides a practical solution for improving the reliability of in situ quality control in hot-forming processes. Future work may involve integrating comprehensive optical models, addressing lens distortions, and extending the framework to accommodate dynamic temperature fields and real-time measurement scenarios. Considering the temporal resolution of the IRIF, it is also possible to differentiate between epistemic and aleatory uncertainties. Future investigations could minimize the aleatory uncertainty contribution and correct the reconstructed point clouds based on the modeled epistemic uncertainties. Although the GPU-based ray tracing approach shows promising results and aligns with the theoretical expectations, a thorough experimental validation with real measurement data is still pending. We plan to conduct controlled experiments under representative hot-forming conditions, capturing camera imagery of heated components and comparing the recorded optical distortions to the simulation’s predictions. This direct comparison will help to quantify the method’s accuracy in real-world scenarios, reveal potential discrepancies caused by unmodeled effects (e.g., turbulence and additional reflections), and provide a solid foundation for further refinements of the simulation framework.

## Figures and Tables

**Table 1:** COMSOL simulation configurations.

Mesh Resolution	Configuration Name	Isosurface Resolution	Configuration Name
Coarse	A	No Refinement	no subfix
Normal	B		
Fine	C	Extra-fine	+
Extra-Fine	D		

**Table 2:** Ray tracer configurations for measurement planning.

Scene		Camera		Ray Tracer	
COMSOL Config.	C	Sensor Size	6.7 mm × 5.6 mm	Evaluation	Yes
Trimming	Yes	Resolution	2448 px × 2048 px	Visualization	Yes
Smoothing	No	Scaling	0.5	Storage	Yes
Cylinder Diameter	27 mm	Focal Length	10 mm		
Cylinder Length	170 mm	Position	Variable		
Cylinder Polygons	2000	Rotation	Variable		

## Data Availability

Dataset available on request from the authors.

## Abbreviations

The following abbreviations are used in this manuscript:

GPU	Graphics Processing Unit
IRIF	Inhomogeneous Refractive Index Field
RMSE	Root Mean Squared Error

## Appendix A. Light Deflection Results

Figure A1a depicts the number of polygons (isosurface triangles) within the camera’s field of view for each tested position. While some positions reveal a larger portion of the mesh (i.e., the mesh appears larger in view), this does not necessarily determine simulation time since additional processes such as building or traversing the bounding volume hierarchy (BVH) also influence overall performance. Figure A1b shows the total number of “ray hits” observed at each camera position, specifically indicating how many of the camera’s viewing rays intersect with the hot object. Although this measure partially correlates with the coverage of the object, it serves as an absolute metric for the number of lines of sight that successfully strike the object. Consequently, camera positions that capture only a small fraction of the object result in fewer ray hits, while more frontal or broader perspectives generate a greater number of hits.

## Appendix B. Histograms of Metrics and Methodology

Figure A2 and Figure A3 present a multi-step, multi-criteria methodology for identifying an optimal camera pose based on several performance metrics. In Figure A2, each metric displacement, coverage, axial resolution, and lateral resolution is illustrated for all evaluated camera positions and normalized to a [0,1] scale. Minimum and near-maximum thresholds (e.g., the 95th percentile) are applied to ensure that otherwise disparate numerical ranges are comparable. Additionally, coverage is inverted so that all metrics adhere to a “lower is better” convention. This process highlights the trade-offs: for instance, a camera pose that excels in minimizing displacement might only achieve moderate resolution or coverage.

In Figure A3, these normalized metrics are combined into a single aggregated score through a weighted summation, enabling users to prioritize specific factors (e.g., low refraction vs. high coverage). A lower overall value in this combined score indicates that the respective pose offers a more balanced solution to the competing demands of minimal refraction-induced displacement, broad coverage, and sufficiently fine spatial resolutions.
